# The impact of narrative writing on empathy, perspective-taking, and attitude: Two randomized controlled experiments on violations of Covid-19 protection regulations

**DOI:** 10.1371/journal.pone.0254501

**Published:** 2021-07-12

**Authors:** Martina Bientzle, Marie Eggeling, Marie Kanzleiter, Kerstin Thieme, Joachim Kimmerle

**Affiliations:** 1 Knowledge Construction Lab, Leibniz-Institut fuer Wissensmedien, Tuebingen, Germany; 2 Department of Psychology, Eberhard Karls University Tuebingen, Tuebingen, Germany; University of Hradec Kralove: Univerzita Hradec Kralove, CZECH REPUBLIC

## Abstract

**Objective:**

Two randomized controlled experiments investigated if writing a narrative text about a fictional person who shows disapproved of behavior in the Covid-19 pandemic influenced empathy, perspective-taking, attitude, and attribution of causes regarding that person’s behavior.

**Methods:**

In both studies, a fictional scenario was described, and participants answered questions regarding empathy, perspective-taking, attitude, and attribution regarding a fictional person’s disapproved of behavior (pre-post-measurement). Participants were randomly assigned to one of two conditions. In the experimental condition, they wrote a narrative text about the fictional person. In the control condition, they wrote about an unrelated topic.

**Results:**

We found that writing a narrative text increased empathy more strongly than writing about an unrelated topic; Study 1: p = 0.004, _part._η^2^ = 0.06, Study 2: p < .001, _part._η^2^ = 0.19. This did not apply to perspective-taking; Study 1: p = 0.415; Study 2: p = 0.074. We also found that writing a narrative text about a fictional person resulted in a more positive attitude toward this person; Study 1: p = 0.005, _part._η^2^ = 0.06; Study 2: p<0.001, _part._η^2^ = 0.10. Finally, in Study 2 we found that participants who wrote a narrative text attributed the person’s behavior to internal causes to a lesser degree; p = 0.007, _part._η^2^ = 0.05.

**Conclusion:**

Our findings indicate that empathy and attitude are positively modifiable through narrative writing tasks. Empathy training could potentially prevent discrimination related to Covid-19.

**Trial registration:**

The studies presented in this article were pre-registered on the pre-registration platform *AsPredicted* (aspredicted.org) before we began data collection; registration numbers and URL: #44754 https://aspredicted.org/vx37t.pdf (Study 1), and #44753 https://aspredicted.org/ig7kq.pdf (Study 2).

## Introduction

The consequences of the Covid-19 pandemic have been a worldwide challenge for more than a year now. To control the spread of the virus and protect people’s health, governments introduced different restrictions and regulations, for example, limiting contact and wearing face masks. The perception of and adherence to these regulations differ a lot across individuals, societies, and countries. An important characteristic of the restrictions to prevent the spread of Covid-19 is that violations can have adverse health effects not only for offenders themselves but also for others. Behavior that is actively harmful to health is generally stigmatized by society and thus triggers prejudice [1]. Drug abuse is an example of such stigmatized behavior. Specific diseases, such as AIDS, obesity, or mental illness can also lead to stigmatization [2,3]. Therefore, it is no surprise that just being sick from Covid-19 already has a stigma attached to it [3]. One explanation for the stigmatization is that observing behavior associated with ill health can trigger strong negative feelings and reactions and consequently lead to negative attitudes toward individuals who exhibit this behavior [4]. The more strongly people are critically perceived to be responsible for their own behavior, the less empathy is shown toward them [5]. Especially in times marked by fear and the threat of a virus, a punitive and intolerant attitude toward others can be observed [6].

People try to understand why others behave the way they do, especially when it comes to critical or stigmatized behavior. The attribution of causes for that behavior helps to classify situations correctly in everyday life and to be able to react appropriately [7]. However, when evaluating behavior, especially disapproved of behavior of others, people underestimate the role of external, situational factors. They tend instead to attribute the negative behavior to internal causes, that is, to the other person’s personality [8], a phenomenon referred to as the *fundamental attribution error* [9].

### Perspective-taking and empathy

Attribution errors can be revised in subsequent information-processing steps [10]. However, these reconsiderations do not happen easily, as they require sufficient levels of attention, motivation, and effort. Taking the perspective of another person can enable those required processes and is thus associated with improving one’s attitude toward the other person [11] and reducing attribution errors [12]. Perspective-taking comprises the active consideration of other people’s mental conditions and their subjective experience. Perspective-taking includes both cognitive and affective mechanisms [13]. Empathy, as an affective component, can have its effect through two different mechanisms: Parallel empathy implies experiencing the same emotion as the target person, that is, one feels as that other person; reactive empathy entails feeling for another with emotional concern for that person’s well-being. Perspective-taking can be effective through both types of empathic responses. Putting oneself in the situation of a person who shows disapproved of health behavior may increase empathetic concern, especially when focusing on that person’s individual feelings [14]. As for cognitive mechanisms, a number of modes of action are known. We refer here particularly to one of those modes as a shift in attributional thinking. This means that people who engage in perspective-taking tend to assign greater importance to non-dispositional than to dispositional aspects.

Previous research has shown that empathy is related to lower levels of aggression, increased prosocial helping behavior [15], and to increased well-being [16]. Former research has also found that empathy and perspective-taking training can improve attitudes toward stigmatized groups like Syrian refugees [17], Romani people [18], immigrants [19], AIDS patients, drug users, or foreigners [20,21]. Perspective-taking can also durably reduce transphobia [22] and racist bias in a clinical context [23].

We argue that a positive basic attitude even toward people who behave problematically is useful in bringing about positive changes in behavior. Stigmatization otherwise tends to increase alienation and divide society further. Devaluation is unlikely to win a person over to different health behavior.

### Narrative writing to reduce stigmatization

Narrative or creative writing has been shown to be effective as one intervention for increasing empathy and perspective-taking in training sessions [24–26]. Unlike non-fictional texts, narrative texts are not about generalizable facts but about specific, individual peculiarities of circumstances, situations, or people. During a narrative writing intervention, participants usually write a short text or story about one or more fictional characters and these characters’ traits, personal experiences, and relationships to others [27]. In their systematic review, Milota and colleagues [28] found that narrative writing training for medical students led to greater empathy [29,30] and a better awareness of patients’ perspectives [31]. Possible reasons for the effectiveness of narrative writing for increasing empathy and perspective-taking are the identification with a character and the reflection on their possible emotions. Narrative writing has also demonstrated social relevance for current situations, in that it can reduce political polarization [32]. To assist inexperienced writers, the writing assignment can be preceded by character development to engage the writer with the person being described [33].

### Research questions and hypotheses

We conducted two experiments to investigate whether writing a narrative text about a fictional person who shows disapproved of behavior in response to the Covid-19 pandemic influenced empathy, perspective-taking, attitude, and attribution of causes regarding that person’s behavior. In Study 1, the disapproved of behavior consisted of violating restrictions by going to work with typical Covid-19 symptoms; in Study 2, the disapproved of behavior was violating restrictions by not wearing a mask on the train. In both studies, participants were put into a fictional situation and wrote either about the fictional person (experimental condition) or about an unrelated topic (in both studies about the room where they stayed during the experiment; control condition). Given the confluence of perspective-taking and empathy described above [13], we stated the following hypotheses:

*Hypothesis 1*. Writing a narrative text about a fictional person will increase empathy (H1a) and perspective taking (H1b) more strongly than writing about an unrelated topic.
*Hypothesis 2*. Writing a narrative text about a fictional person will result in a more positive attitude toward this person than writing about an unrelated topic.
*Hypothesis 3*. Participants who write a narrative text about a fictional person will attribute that person’s behavior to a lesser degree to internal causes than participants who write about an unrelated topic.

As an open research question, we investigated if the intervention had an impact on individual attitudes toward Covid-19 protection restrictions in general.

## Materials and methods

### Ethical approval

The studies presented here were part of a research project that was approved by the Ethics Committee of the Leibniz-Institut für Wissensmedien (approval number: LEK 2020/032).

### Sample

Power analysis for ANOVAs with α = 0.05, an intended power of 95%, and a medium effect size of f = 0.25 revealed a required sample size of N = 158 for each experiment.

We excluded participants who (1) indicated that they were not adequately motivated to participate in the study, (2) did not have adequate German language skills, or (3) indicated that the recommendation not to go to work with typical Covid-19 symptoms/to wear a face mask while riding a train was unnecessary. This last exclusion criterion was implemented because this study was about how people dealt with disapproved of behavior, and therefore we only wanted to include participants in the sample who actually disapproved of this behavior. We also did not invite participants who were younger than 18 years old or who did not speak German fluently.

N = 1878 potential participants on the online participant recruitment platform *Prolific* (https://www.prolific.co) fulfilled our inclusion criteria and were invited to participate in the studies. Data collection for both experiments was conducted at the same time, and after accepting the invitation, participants were randomly assigned to either Study 1 or Study 2.

N = 155 people started participating in Study 1; 21 participants had to be excluded from the data analysis because they had not given their consent (n = 1), canceled the survey early (n = 9), indicated that they had not been adequately motivated to participate in the study (n = 6), or indicated that the recommendation not to go to work with typical Covid-19 symptoms was unnecessary (n = 5). After these exclusions, the data from N = 134 participants (experimental condition: n = 65 participants; control condition: n = 69 participants) were analyzed. There were no group differences regarding gender (female: n = 53; male: n = 79; diverse: n = 2; χ^2^ = 2.218, p = 0.330) or age (M = 28.77, SD = 8.90; t(132) = 1.884, p = 0.062).

N = 158 people started participating in Study 2; 22 participants had to be excluded from the data analysis because they had not given their consent (n = 3), canceled the survey early (n = 11), indicated that they had not been adequately motivated to participate in the study (n = 4), or indicated that the recommendation to wear a face mask when riding a train was unnecessary (n = 4). After these exclusions, the data from N = 136 participants (experimental condition: n = 66 participants; control condition: n = 70) were analyzed. There were no group differences regarding gender (female: n = 65; male: n = 71; χ^2^ = 0.250, p = 0.617) or age (M = 30.70, SD = 9.54; t(134) = -0.24, p = 0.815).

**Fig 1:**
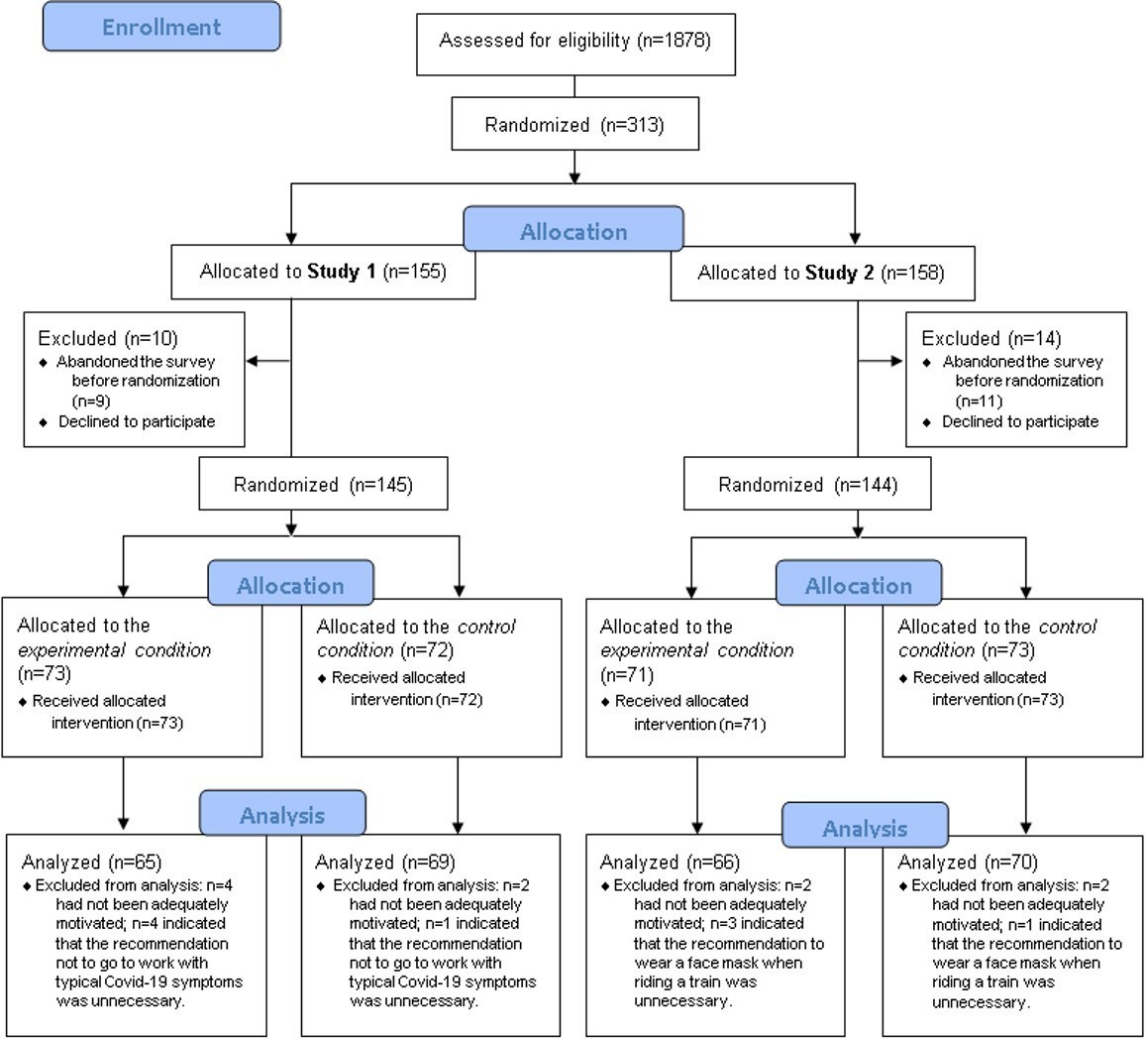
Sampling procedure.

A detailed overview of the sampling procedure can be seen in Fig 1.

### Procedure

The instructions as well as the design and the dependent measures were adopted from a study by Shaffer and colleagues [28]. The experiments were conducted online, using the participant recruitment platform Prolific and the online tool *Qualtrics* Survey Software [34]. *Qualtrics* Survey Software performed the randomized assignment of participants to conditions based on a computer-controlled random generator without human intervention. Before starting the survey, participants provided their written informed consent. Then the fictional scenario was described, after which participants answered questions regarding empathy, perspective-taking, attitude, and attribution of causes regarding the fictional person’s behavior. Next, participants were randomly assigned to one of two conditions. In the experimental condition, they received the instructions to write a narrative text about the fictional person who showed the disapproved behavior. To prepare for this task, they were instructed to imagine the fictional person in a concrete way and think about possible answers to questions regarding the person (e.g., name, age, living situation). The only constraint was that the fictional person should be regarded to be at least as intelligent as the participants themselves. Then participants were asked to take a minimum of ten minutes to write their scene. In the control condition, participants received the instructions to write for ten minutes about the room they were staying in during the experiment. An automatic timer in the survey prevented them from continuing to the next page before this writing time had passed. After finishing the writing task, participants answered again the same questions regarding empathy, perspective-taking, attitude, and attribution. In addition, they were asked for their own opinion about the specific violation in question, their motivation while taking part in the study, and demographic data (age and gender). As an exploratory measure they were asked for their attitude toward Covid-19 protection restrictions in general.

### Material

Shaffer and colleagues [35] used the fictional scenario of seeing a pregnant woman smoking a cigarette in a parking lot in front of a supermarket. The studies presented here aimed at replicating and extending the findings of Shaffer and colleagues [35] by using typical situations where people show adverse health behavior in the context of the current Covid-19 pandemic, and in doing so exhibit behavior that may also be potentially harmful to the health of others.

The fictional scenario for Study 1 was: “You enter a bakery. The saleswoman serving you shows symptoms typical of a Coronavirus infection. She coughs strongly and looks feverish.”

The fictional scenario for Study 2 was: “You are traveling by train. While doing so, you notice that a female railroad employee is not wearing a face mask during a ticket inspection, despite the Coronavirus protection regulations.”

### Measures

All dependent variables were assessed twice: after reading the description of the situation (t1) and after writing the text (t2).

*Empathy* and *perspective-taking* were measured using an adapted version of the Saarbrückener Persönlichkeitsfragebogen (SPF) [36]. The SPF is the German version of the Interpersonal Reactivity Index (IRI) [37], consisting of the four subscales *perspective-taking*, *empathic concern*, *fantasy*, and *personal distress*. As in the study by Shaffer and colleagues [35], we used only the subscales *perspective-taking* and *empathic concern*. The four perspective-taking subscale items measure the ability to cognitively put oneself in the other person’s position, while the four items of the empathic concern subscale measure emotional involvement in the feelings of others. The items were adapted to the fictional scenarios used in our experiments (Table 1). Participants were asked to indicate their agreement to the statements on a scale from “don’t agree at all” (0) to “fully agree” (100). Internal consistency scores for the *perspective-taking* scale were excellent (Cronbach alpha in Study 1 at t1: *α* = 0.93 and t2: *α* = 0.90; Cronbach alpha in Study 2 at t1: *α* = 0.90 and t2: *α* = 0.92); internal consistency scores for the *empathy* scale were acceptable to good (Cronbach alpha in Study 1 at t1: *α* = 0.81 and t2: *α* = 0.89; Cronbach alpha in Study 2 at t1: *α* = 0.68 and t2: *α* = 0.88). All items are shown in Table 1.

**Table 1:** Measurement of perspective-taking and empathy.

Perspective-taking	Empathy
I tried to imagine her perspective of the problem.	I felt the need to protect the bakery saleswoman/the railroad employee.
I tried to look at the bakery saleswoman’s/railroad employee’s perspective in addition to my own.	Just imagining the situation has emotionally touched me.
Before I criticized the bakery saleswoman/the railroad employee, I tried to imagine how I would feel if I were in her place.	I would consider myself a pretty soft-hearted person, based on how I felt when I imagined the scene.
When the bakery saleswoman’s/the railroad employee’s behavior seemed strange to me, I tried to put myself in her shoes.	I felt warm feelings for the bakery saleswoman/the railroad employee.

*Attitude* was measured with a feeling thermometer [38]. Feeling thermometers are frequently used to measure feelings and attitudes toward individuals or groups [39]. This attitude measure was used analogously to a thermometer that measures temperature and allowed the attitude toward the fictional person to be ranked on a visual-analog scale of 0–10. Zero symbolized a very “cold” or negative attitude toward the fictional person, the value 5 corresponded to a neutral attitude, and a value of 10 symbolized a very “warm” or positive attitude.

*Attribution* was measured with three single items (see Table 2), following the procedure used by Shaffer and colleagues [35]. Participants were again asked to indicate their agreement with the statements on a scale from “not at all” (0) to “very much” (100).

**Table 2:** Measurement of attribution.

Item 1	To what extent is this person to blame for her actions?
Item 2	To what extent are environmental factors, such as life circumstances, responsible for this person’s behavior?
Item 3	To what extent does the bakery saleswoman/the railroad employee have the freedom to make better choices?

The exploratory measure *attitude toward the Covid-19 protection restrictions* was measured at t2 with ten items adapted from the Risk-Taking Attitude and Risky Driving Behavior questionnaire [40] (see Table 3). Participants were asked to indicate their agreement with the statements on a scale from “don’t agree at all” (0) to “fully agree” (100). Internal consistencies for the scale were good (Cronbach alpha in Study 1: *α* = 0.81; Cronbach alpha in Study 2: *α* = 0.87).

**Table 3:** Measurement of attitude toward the Covid-19 protection restrictions.

1^r^	Many pandemic regulations have to be ignored in order to make social life possible.
2	Pandemic regulations must be accepted irrespective of the situation.
3^r^	The pandemic regulations are not respected because they are too restrictive.
4^r^	Violating a few pandemic regulations does not make you a bad person.
5^r^	It is okay to break the pandemic regulations if no other person is directly involved.
6^r^	The pandemic regulations are often too complicated to implement them all.
7^r^	If you are healthy, it is okay not to always follow the pandemic regulations.
8	The penalties for rule violations should be more severe.
9^r^	It is okay to be out with someone who does not follow the pandemic regulations if others do so as well.
10	I do not want to risk my health by being out with others who do not follow the pandemic regulations.

^**r**^ Indicates reversely coded items.

At the end of the survey, participants responded to three items regarding their motivation to answer the questions honestly and carefully, and one item regarding their attitude toward the demand not to go to work with typical Covid-19 symptoms/to wear a face mask in public.

### Analysis

Data analysis was performed using IBM SPSS 25 statistics for Windows. Normal distribution was not given for most variables. Therefore, we performed analyses of variance (ANOVA), including repeated measure analysis to test our hypotheses, since simulation studies have shown that ANOVAs are robust to violations of the normal distribution assumption [41,42]. We provide means (M) and standard deviations (SD) as well as F-values, p-values, and partial eta-squared (_part._ η^2^) as an indicator of effect size.

## Results

### Empathy and perspective-taking

**Fig 2:**
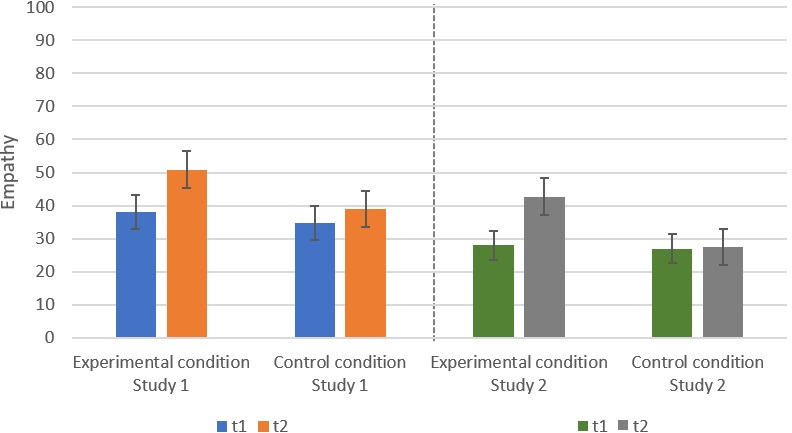
Significant interaction effects between time of measurement and condition regarding empathy. Confidence intervals are represented by the error bars attached to each column.

There was a significant increase in empathy for the participants in both studies across both conditions; Study 1: F(1, 132) = 33.95, p < .001, _part._ η^2^ = 0.21; Study 2: F(1, 134) = 36.95, p < .001, _part._ η^2^ = 0.22. At t1, participants indicated less empathy (Study 1: M = 36.34, SD = 21.34; Study 2: M = 27.48, SD = 18.27) than at t2 (Study 1: M = 44.74, SD = 23.25; Study 2: M = 34.90, SD = 24.14). In Hypothesis 1a, we had stated that writing a narrative text about a fictional person would increase empathy more strongly than writing about an unrelated topic. The data of both studies supported this hypothesis (Fig 2 and Table 4); Study 1: F(1, 132) = 8.49, p = 0.004, _part._ η^2^ = 0.06, Study 2: F(1,134) = 32.10, p<0.001, _part._ η^2^ = 0.19.

**Table 4:** Means and standard deviations of the outcome variables in the experimental and the control conditions.

			Study 1		Study 2	
			M	SD	M	SD
**Empathy**	Experimental condition	t1	38.11	21.97	27.99	27.98
		t2	50.90	24.29	42.72	23.56
	Control condition	t1	34.67	20.76	27.01	19.35
		t2	38.93	20.76	27.53	22.44
**Perspective taking**	Experimental condition	t1	59.30	26.20	52.42	26.55
		t2	71.43	19.32	66.76	22.58
	Control condition	t1	53.28	25.84	50.51	28.34
		t2	62.37	22.40	58.06	27.30
**Attitudes toward the fictional character**	Experimental condition	t1	2.57	1.98	2.12	1.71
		t2	4.59	2.62	4.06	2.40
	Control condition	t1	2.25	1.87	2.36	2.05
		t2	3.35	2.05	3.00	2.32
**Attribution: Item 1**	Experimental condition	t1	68.52	22.09	75.68	18.52
		t2	60.62	27.78	67.67	24.43
	Control condition	t1	71.88	20.32	78.09	24.95
		t2	67.90	21.86	74.56	74.56
**Attribution: Item 2**	Experimental condition	t1	67.29	24.37	46.80	26.95
		t2	70.28	23.68	53.83	28.00
	Control condition	t1	66.36	21.26	44.29	27.02
		t2	65.64	23.41	51.07	28.11
**Attribution: Item 3**	Experimental condition	t1	63.17	27.46	79.35	23.13
		t2	56.71	29.45	73.94	23.65
	Control condition	t1	69.49	21.95	67.09	32.77
		t2	67.74	21.91	69.51	29.89
**Attitudes toward the Covid-19 protection restrictions**	Experimental condition		64.93	13.95	69.34	15.20
	Control condition		67.86	14.25	72.41	17.12

There was also a significant increase in perspective-taking for the participants in both studies across both conditions; Study 1: F(1, 132) = 32.36, p < .001, _part._ η^2^ = 0.20; Study 2: F(1, 134) = 33.88, p < .001, _part._ η^2^ = 0.20. At t1, they indicated a lower score in perspective-taking (Study 1: M = 56.20, SD = 26.10; Study 2: M = 51.44, SD = 27.40) than at t2 (Study 1: M = 66.76, SD = 21.37; Study 2: M = 62.28, SD = 25.41). Contrary to Hypothesis 1b, there were no significant interaction effects of time and condition in Study 1 (F(1, 132) = 0.67, p *=* 0.415, _part._ η^2^ = 0.005) or in Study 2 (F(1, 134) = 3.25, p *=* 0.074, _part._ η^2^ = 0.02). Writing a narrative text about a fictional person did not increase perspective-taking any more strongly than writing about an unrelated topic.

### Attitude

**Fig 3:**
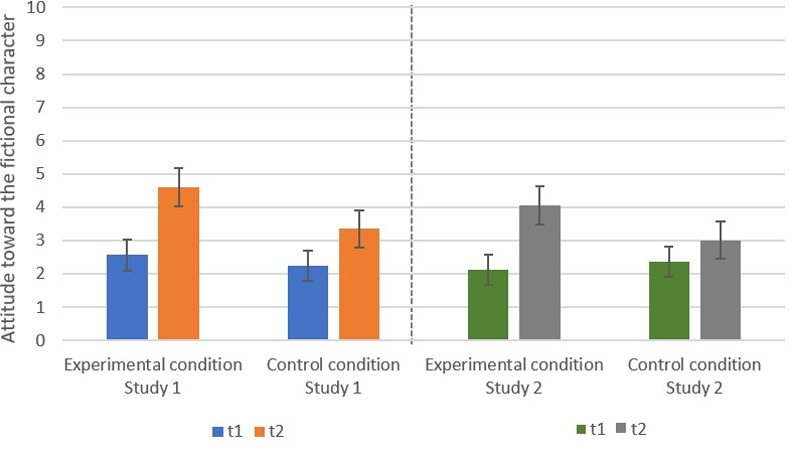
Significant interaction effects between time of measurement and condition regarding attitude toward the fictional character. Confidence intervals are represented by the error bars attached to each column.

Again, we found a significant effect of time in both studies; Study 1: F(1, 132) = 93.86, p < .001, _part._ η^2^ = 0.42; Study 2: F(1, 134) = 61.47, p < .001, _part._ η^2^ = 0.31. At t1, the attitude was more positive (Study 1: M = 3.95, SD = 2.41; Study 2: M = 3.51, SD = 2.41) than at t2 (Study 1: M = 2.40, SD = 1.92; Study 2: M = 2.24, SD = 1.89) across both conditions. We stated in Hypothesis 2 that writing a narrative text about a fictional person would result in a more positive attitude toward this person than writing about an unrelated topic. The data of both studies supported this assumption: Study 1: F(1, 132) = 8.07, p *=* 0.005, _part._ η^2^ = 0.06; Study 2: F(1, 134) = 15.50, p < .001, _part._ η^2^ = 0.10 (Fig 3).

### Attribution

We observed significant pre-post differences regarding the first attribution item in both studies (Study 1: F(1, 132) = 22.25, p<0.001, _part._ η^2^ = 0.14; Study 2: F(1, 134) = 10.91, p *=* 0.001, _part._ η^2^ = 0.08) but no significant interaction effects (Study 1: F(1, 132) = 2.42 p = 0.122, _part._ η^2^ = 0.02; Study 2: F(1, 134) = 1.65 p *=* 0.201, _part._ η^2^ = 0.01). This showed that participants were less likely to believe that the fictional person was to blame for her disapproved of health behavior after writing the text (Study 1: M = 64.37, SD = 25.08; Study 2: M = 71.21, SD = 24.54) than before (Study 1: M = 70.25, SD = 21.18; Study 2: M = 76.92, SD = 22.02), regardless of whether they wrote about the person or an unrelated topic.

Only in Study 2 was there a significant pre-post effect regarding the second attribution item (Study 1: F(1, 132) = 0.561, p = 0.455, _part._ η^2^ = 0.004; Study 2: F(1, 134) = 16.59, p < .001, _part._ η^2^ = 0.11). In Study 2, participants were more likely to believe that external factors, such as life circumstances, were responsible for the fictional person’s disapproved of health behavior after writing the text (Study 1: M = 67.89, SD = 23.57; Study 2: M = 52.41, SD = 27.99) than before (Study 1: M = 66.81, SD = 22.74; Study 2: M = 45.51, SD = 26.91). Again, there were no significant interaction effects (Study 1: F(1, 132) = 1.51, p = 0.221, _part._ η^2^ = 0.01; Study 2: F(1, 134) = 0.005, p = 0.943, _part._ η^2^<0.001). We observed that writing a narrative text about a fictional person did not decrease attribution of the negative health behavior to internal causes more strongly than writing about an unrelated topic.

Regarding the third attribution item, there was a significant pre-post effect in Study 1 only (Study 1: F(1, 132) = 9.08, p = 0.003, _part._ η^2^ = 0.06; Study 2: F(1, 134) = 1.10, p = 0.297, _part._η^2^ = 0.008). In Study 1, participants believed to a lesser degree that the fictional person had the freedom to make better choices at t2 (Study 1: M = 62.39, SD = 26.33; Study 2: M = 71.66, SD = 27.04) than a t1 (Study 1: M = 66.43, SD = 24.88; Study 2: M = 73.04, SD = 29.05). In addition, there was a significant interaction effect in Study 2 (Study 1: F(1, 132) = 2.98, p = 0.087, _part._ η^2^ = 0.02; Study 2: F(1, 134) = 7.57, p = 0.007, _part._ η^2^ = 0.05). This effect was consistent with Hypothesis 3, indicating that participants who wrote a narrative text about the fictional person attributed that person’s behavior to a lesser degree to internal causes than participants who wrote about an unrelated topic.

### Attitude toward the Covid-19 protection restrictions

As an open research question, we investigated if the intervention had an impact on the individual attitudes toward the Covid-19 protection restrictions in general. In both studies, we found no significant effect (Study 1: t(132) = -1.201, p = 0.232; Study 2: t(134) = -1.106, p = 0.271).

## Discussion

The studies presented here examined the influence of a narrative writing intervention on empathy, perspective-taking, attitude, and attribution of causes toward a fictional person who exhibited disapproved of health behavior. As expected, empathy and attitude toward the fictional person changed more positively in the experimental group than in the control group. Contrary to the hypotheses and in contrast to the results of Shaffer and colleagues [35], no stronger change in perspective-taking was found in the experimental group compared to the control group. Regarding attribution, the pattern of results was mixed—the result of Shaffer and colleagues [35] was replicated in only one case (Study 2, attribution item 3). Such an inconsistent result should be interpreted with great caution, and even more so since attribution was only measured with single items and not with a validated scale. The results of both studies suggest that empathy and attitude can be modified to become more positive through a narrative writing task. This fits with previous research showing that people can be trained in empathy and attitude specifically and simultaneously [25,35,43] and that even ten to fifteen minutes of writing training can be sufficient for this purpose.

In contrast to the cognitive process of perspective-taking, empathy as an affective component changed more strongly through the narrative writing task in the experimental than in the control group. There are several possible explanations for this finding. It is possible that narrative writing has a stronger effect on an affective level. For example, writing tasks have already been shown in several clinical studies to be effective in minimizing depressive rumination and improving emotion processing [44–46]. In addition, Shapiro and colleagues [26] postulated that creative writing elicits increased emotions. Another explanation could be that perspective-taking is a more complex process and thus requires more time than a ten-minute narrative writing task allows.

The fact that most of the dependent variables in the experimental condition as well as in the control condition changed in a positive direction over time indicates that the writing task of the control condition also induced an effect. Since both conditions involved a ten-minute writing task, perhaps writing a text alone had an effect, because it helped participants focus better on themselves and activate a specific mindset. The participants of the control group described the environment around them. This could have increased their self-awareness and mindfulness. Previous research has shown that even non-emotional narrative writing, in which participants were instructed to write about their daily lives, may increase mindfulness [47]. Whether the activity of writing itself caused the effect cannot be answered in the context of our studies. However, it is rather unlikely that a writing task that does not require any self-awareness would have the same impact on empathy and attitude. Further studies are needed to explain the effects found in these studies.

Even though we found significant changes in the dependent variables in the experimental condition, it is unclear which step of the writing task induced these changes. Before actually writing, participants answered questions on character development, which may have already caused participants to think more deeply about the fictional person. Thinking more deeply about the characters one plans to write about before actually starting to write a text is a normal preparation step. It is possible that either these questions, or the writing task alone, or a combination of these two steps contributed to the changes in the dependent variables, which leaves the question open.

In previous studies, it also remained ambiguous in some cases which factors of the intervention led to particular effects. For example, DasGupta and Charon [24] combined a face-to-face meeting with a writing task. Moreover, narrative medicine training sessions combined reading, writing, and discussing people and situations [48]. In future studies, it would be interesting to investigate which steps exactly are responsible for the changes in perceptions.

Moreover, we cannot rule out the possibility that the effects we observed were experimenter demand effects, at least in part. Participants of the experimental group were asked to write about a person who showed disapproved of behavior in the Covid-19 pandemic before and after answering questions regarding empathy and perspective-taking. Sensing experimenter expectations, the participants might have replied that they were able to feel more empathy toward the person even if they did not actually experience these feelings. However, if our findings were mere experimenter demand effects, perspective-taking and empathy should show the same pattern of results, which was not the case.

Finally, our procedure of excluding observations on the basis of post-treatment criteria can be criticized [49]. We excluded participants who indicated that they were not adequately motivated to participate in the study and who assessed the Covid-19 restrictions addressed in the studies as unnecessary. It is possible that these criteria were influenced by the treatment, which in turn could lead to a posttreatment bias. In future studies, these variables should be collected before treatment implementation.

## Conclusion

Empathy, perspective-taking, and attitude toward patients play a significant role in social interactions and especially in the healthcare system [50,51]. Therefore, for a fair healthcare system and equal treatment of all patients, investment should be made in empathy training for healthcare workers. Our findings indicate that empathy and attitude can be modified in a positive direction through even a narrative writing task that does not take much time. The present studies as well as other recent work on Covid-19 [3,52,53] showed that new socially stigmatized behaviors are emerging due to the Covid-19 pandemic. Empathy training could potentially address this occurrence to prevent early stigma and discrimination related to Covid-19.

### Practice implications

For educational institutions in the medical context, but also for schools or other training institutes, narrative writing could be an efficient and creative soft-skill method to strengthen interpersonal relationships and community empathy. Narrative writing has also demonstrated current social relevance, in that it can reduce political polarization [32]. In contrast to consuming audio-visual media, writing necessarily requires an active and mindful engagement with the subject matter.
